# Real-Time Non-Uniformity Correction Method for 800 FPS High-Frame-Rate Short-Wave Infrared Images

**DOI:** 10.3390/s26072209

**Published:** 2026-04-02

**Authors:** Guiguang Su, Yueming Wang, Daogang He

**Affiliations:** 1 Hangzhou Institute for Advanced Study, University of Chinese Academy of Sciences, Hangzhou 310024, China; suguiguang23@mails.ucas.ac.cn; 2 University of Chinese Academy of Sciences, Beijing 100049, China; 3 Key Laboratory of Space Active Opto-Electronics Technology, Shanghai Institute of Technical Physics, Chinese Academy of Sciences, Shanghai 200083, China; hedaogang@mail.sitp.ac.cn; 4 Shanghai Key Laboratory of Optical Coatings and Spectral Modulation, Shanghai Institute of Technical Physics, Chinese Academy of Sciences, Shanghai 200083, China

**Keywords:** non-uniformity correction, high frame rate system, quadratic polynomial, infrared imaging, FPGA

## Abstract

In conventional infrared imaging systems, non-uniformity correction typically involves continuously reading correction parameters from double data rate (DDR) memory. For high-frame-rate short-wave infrared imaging systems to achieve real-time non-uniformity correction, it is essential to minimize the reading time of correction parameters. Due to the narrow dynamic range of two-point correction and the large parameter storage required by two-point multi-segment correction, it is difficult to simultaneously achieve good correction performance and short parameter reading time under limited hardware resources. To address the above issues, this paper proposes a real-time non-uniformity correction method suitable for high-frame-rate short-wave infrared images. Based on a field-programmable gate array (FPGA), improvements are made to quadratic polynomial correction through the design of quantization methods for different parameters to enhance storage bit-width utilization; dynamic allocation of bit-widths between parameters to improve correction performance; and ping-pong buffering for DDR reading to avoid the impact of DDR read latency on parameter reading time. The storage size of the improved correction parameters is comparable to that of conventional two-point correction. Experiments were conducted on a hardware system based on the XC7A100T-2FGG484I FPGA. The experimental results show that the average non-uniformity of images after the improved quadratic polynomial correction is 0.4818%, significantly better than 0.5930% after two-point correction and slightly better than 0.4891% after two-point eight-segment correction. Blind pixel compensation was completed simultaneously with the correction. Using a 640 × 512 area array InGaAs short-wave infrared detector, the highest real-time processing frame rate reaches 800 frames per second (FPS).

## 1. Introduction

Nowadays, high-frame-rate infrared imaging systems play a crucial role in numerous fields, including high-speed missile infrared seekers, multispectral imaging technology, and laser communication [1,2]. When uniform external radiation is input into an infrared imaging system, due to the inherent characteristics and manufacturing processes of the infrared focal plane detector, the pixels in the focal plane exhibit response characteristics that differ from those of adjacent pixels [3]. This variation in response characteristics is also known as non-uniformity. The presence of non-uniformity severely affects the imaging quality and radiation measurement accuracy of infrared imaging systems [4]. High-frame-rate infrared systems inevitably require processing of non-uniformity in infrared images. Given the extremely high data transmission rates in high-frame-rate systems, achieving better non-uniformity reduction while enabling real-time non-uniformity correction is often mutually exclusive. Therefore, research on real-time non-uniformity correction for high-frame-rate imaging systems holds significant importance.

Currently, two-point correction is the most widely used method in practical applications [5]. The research team led by Cheng et al. [6] has applied two-point correction to high-frame-rate infrared systems, achieving real-time image output at 200 FPS with an array size of 320 × 256. Meanwhile, the team led by Wang et al. [7] and Han et al. [8] improved two-point correction by combining it with single-point correction without increasing computational complexity, resulting in better correction performance than conventional two-point correction. Two-point correction is computationally simple, requires minimal storage resources, and is easy to implement, making it often the optimal choice under limited hardware resources. However, two-point correction assumes linear response from the infrared detector pixels, whereas actual responses often exhibit nonlinearity. Thus, its performance is limited by the selection of calibration points and cannot balance local and global correction performance, reflecting that two-point correction is not suitable for wide dynamic range applications [9]. To overcome the shortcomings of two-point correction, the team led by Wang et al. [10] improved it by proposing multi-point correction, also known as piecewise two-point correction, which uses a polyline model to approximate the actual response of the infrared detector. The more calibration points, the higher the accuracy of multi-point correction [11]. However, in addition to performing one multiplication and addition operation per pixel like two-point correction, multi-point correction requires multiple comparison operations for each pixel value [12]. As the number of calibration points increases, the number of comparison operations also rises, consuming substantial storage resources for multi-segment correction parameters. Although multi-point correction retains the computational simplicity of two-point correction while applying to a wider dynamic range, its numerous comparison operations and large storage resource consumption make it unsuitable for high-frame-rate infrared imaging systems. Besides calibration-based two-point and multi-point corrections, there are also scene-based non-uniformity correction algorithms. Scene-based correction algorithms can be roughly divided into four categories [13]: temporal high-pass filter (THPF) methods [14], constant statistics methods [15,16,17], neural network methods [18], and registration-based methods [19]. These scene-based correction methods can adapt to non-uniformity changes caused by temperature drift and time drift in devices, avoiding reliance on calibration. However, scene-based methods are highly dependent on the scene; if the scene is static or lacks motion, it may lead to slow convergence of fixed pattern noise or even algorithm failure. Moreover, most scene-based correction methods involve high computational loads, and excessive computational delays make it difficult to meet the real-time requirements of algorithms in high-frame-rate imaging systems [20].

This paper analyzes the characteristics of two-point correction and multi-point correction, and, in combination with the requirements of high-frame-rate infrared imaging systems [21], improves the quadratic polynomial correction based on an FPGA. By designing quantization methods for different parameters and dynamically allocating storage bit-widths among parameters, the proposed method significantly enhances storage bit-width utilization and correction performance while limiting the storage of each pixel’s correction parameters to only 32 bits. This effectively overcomes the limitations of the narrow dynamic range in conventional two-point correction and the large parameter storage required by two-point multi-segment correction. Furthermore, a ping-pong buffering mechanism is adopted for reading DDR parameters, which effectively hides the impact of DDR read latency on parameter reading time and enables real-time matching between correction parameters and image data under high-frame-rate conditions. By constructing a high-frame-rate short-wave infrared experimental platform, the correction performance and real-time processing capability of the proposed method at 800 FPS are verified. Blind pixel compensation is also completed simultaneously. The experimental results demonstrate that the correction performance approaches the theoretical level of quadratic polynomial correction, outperforms the two-point eight-segment correction, and the maximum real-time processing frame rate reaches 800 FPS.

## 2. Improved Quadratic Polynomial Correction

### 2.1. Principle of Quadratic Polynomial Correction

In the field of infrared image processing, quadratic polynomial correction serves as a nonlinear compensation method primarily designed to optimize the nonlinear characteristics of the detector response curve. By introducing a quadratic term, this method effectively models the nonlinear deviation between the actual pixel response and the ideal response, thereby achieving more accurate non-uniformity correction under high dynamic range or complex radiation conditions. Compared to linear correction methods, its superiority lies in better adaptation to the detector’s nonlinear response, resulting in improved image quality and radiometric accuracy.

The least squares method is the core technique for estimating the parameters in quadratic polynomial correction. It fits the pixel response curve by minimizing the sum of squared residuals. Assuming the raw digital number (DN) value of a pixel is denoted as x, and the corrected DN value as y, the quadratic polynomial model can be expressed as:

(1) $$y=ax^{2}+bx+c,$$

where a, b, and c are the parameters to be determined, representing the quadratic correction coefficient, the linear correction coefficient, and the constant term, respectively. The fitting process is based on multiple sets of calibration data points (x_i_, y_i_) (i = 1, 2, …, n; where n corresponds to different irradiance/radiance levels). The objective is to minimize the sum of squared residuals. The formula for the sum of squared errors to be minimized is:

(2) $$S=\sum_{i=1}^{n}(y_{i}-(ax_{i}^{2}+bx_{i}+c){)}^{2}$$

To solve for a, b, and c, we take the partial derivatives of S with respect to each parameter and set them to zero, yielding the following system of normal equations:

(3) $$\left\{\begin{array}{c} \frac{\partial S}{\partial a}=-2\sum_{i=1}^{n}(y_{i}-ax_{i}^{2}-bx_{i}-c)x_{i}^{2}=0\\ \frac{\partial S}{\partial b}=-2\sum_{i=1}^{n}(y_{i}-ax_{i}^{2}-bx_{i}-c)x_{i}=0\\ \frac{\partial S}{\partial c}=-2\sum_{i=1}^{n}(y_{i}-ax_{i}^{2}-bx_{i}-c)=0 \end{array}\right.$$

After simplification, the system of equations becomes:

(4) $$\left\{\begin{array}{c} a\sum x_{i}^{4}+b\sum x_{i}^{3}+c\sum x_{i}^{2}=\sum y_{i}x_{i}^{2}\\ a\sum x_{i}^{3}+b\sum x_{i}^{2}+c\sum x_{i}=\sum y_{i}x_{i}\\ a\sum x_{i}^{2}+b\sum x_{i}+cn=\sum y_{i} \end{array}\right.$$

This system of equations can be transformed into matrix form as:

(5) $$\left[\begin{array}{ccc} \sum x_{i}^{4} & \sum x_{i}^{3} & \sum x_{i}^{2}\\ \sum x_{i}^{3} & \sum x_{i}^{2} & \sum x_{i}\\ \sum x_{i}^{2} & \sum x_{i} & n \end{array}\right]\left[\begin{array}{c} a\\ b\\ c \end{array}\right]=\left[\begin{array}{c} \sum y_{i}x_{i}^{2}\\ \sum y_{i}x_{i}\\ \sum y_{i} \end{array}\right]$$

This system of equations can be solved by matrix inversion to obtain the parameter vector [a, b, c]^T^.

### 2.2. FPGA-Based Improvement of Quadratic Polynomial Correction

To adapt to the real-time requirements of high-frame-rate infrared image processing and address the constraints of hardware resources, this paper proposes an improved quadratic polynomial correction method based on an FPGA. This method achieves adaptation of quadratic polynomial correction to high-frame-rate systems through the design of different parameter quantization methods, dynamic allocation of storage bit-widths among parameters, and optimized design of the correction computation pipeline. The core of the improvement lies in balancing correction accuracy with hardware resource consumption, ensuring efficient storage and computation of correction parameters for each pixel within limited bit-widths.

The parameter calculation process first involves fitting and optimization of the collected calibration data, including blind pixel detection and quantization with bit-width allocation for the parameters a, b, and c.

First, the blind pixel flag is marked. The 3σ algorithm [22] is applied to the raw images acquired by the system for blind pixel detection. In a static scene with uniform irradiance incidence, multiple frames of raw images are continuously collected, and a low-noise calibration image is obtained through temporal averaging. Subsequently, global 3σ detection using a full-frame window is performed on the calibration image to calculate the mean and standard deviation of the entire image. If the deviation of a pixel’s DN value from the global mean exceeds three times the standard deviation, the pixel is marked as a blind pixel and its corresponding blind pixel flag is set to 1; otherwise, it is set to 0.

Next, the least squares method is used to fit the collected data at different irradiance levels, thereby calculating the correction parameters a(i, j), b(i, j), and c(i, j) for each pixel, where i and j represent the row and column indices of the pixel.

Subsequently, parameter quantization and bit-width allocation are performed. Since the computed correction parameters are in floating-point format, and considering the advantages of fixed-point arithmetic on an FPGA, the floating-point coefficients are converted to fixed-point format.

The values of parameter a are typically on the order of 10^−6^ to 10^−8^. Direct fixed-point representation would require a large number of bits. Therefore, a magnification approach is proposed for parameter a: all pixels’ a parameters are multiplied by 2^N^, where the magnification factor N is determined by the following Equation (6):

(6) $$N=\left\lfloor \log_{2}\frac{1}{{\left|a(i,j)\right|}_{\max}}\right\rfloor ,$$

where |a|_max_ is the maximum absolute value among all quadratic correction coefficients a(i, j) (the a parameters of blind pixels are excluded from the comparison). The amplified a parameter can be expressed as:

(7) $$a_{N}=a\times 2^{N}$$

The purpose of multiplying by 2^N^ is twofold:

1. To amplify |a(i, j)| as much as possible, thereby making full use of the limited bit-width and representing the amplified a parameter using fixed-point fractional notation;
2. Division operations consume significant resources on an FPGA, whereas scaling the parameter back to its original value dividing by 2^N^ can be implemented simply and efficiently by performing a right shift in N bits. Right-shift operations have extremely low cost and are very easy to implement in FPGA hardware.

The improved quadratic polynomial correction equation implemented on an FPGA is:

(8) $$y=a_{N}x^{2}/2^{N}+bx+c$$

The parameter b has values close to 1 and is represented using an unsigned fixed-point fractional format, with 1 bit allocated for the integer part and the remaining bits for the fractional part.

For parameter c, the specific bit-width is determined based on the maximum and minimum values of c across all pixels (the c parameters of blind pixels are excluded from the comparison). It is represented as a signed integer, with the fractional part rounded to the nearest integer and only the integer part retained.

The storage arrangement of the parameters for each pixel is shown in Table 1.

From Table 1, it can be seen that the storage bit-width for the correction parameters of each pixel is set to 32 bits, of which 1 bit is used for the blind pixel flag, and the remaining 31 bits are allocated to the correction parameters. The specific bit-width allocation for a_N_ and b is determined based on the overall correction performance on the validation dataset. Once the bit-widths for each parameter are finalized, a binary file is generated in row-major order. The parameters are stored sequentially on a per-row basis, which facilitates row-by-row reading by the FPGA.

## 3. Design of a Real-Time Non-Uniformity Correction System for High-Frame-Rate Short-Wave Infrared Images

**Figure 1:**
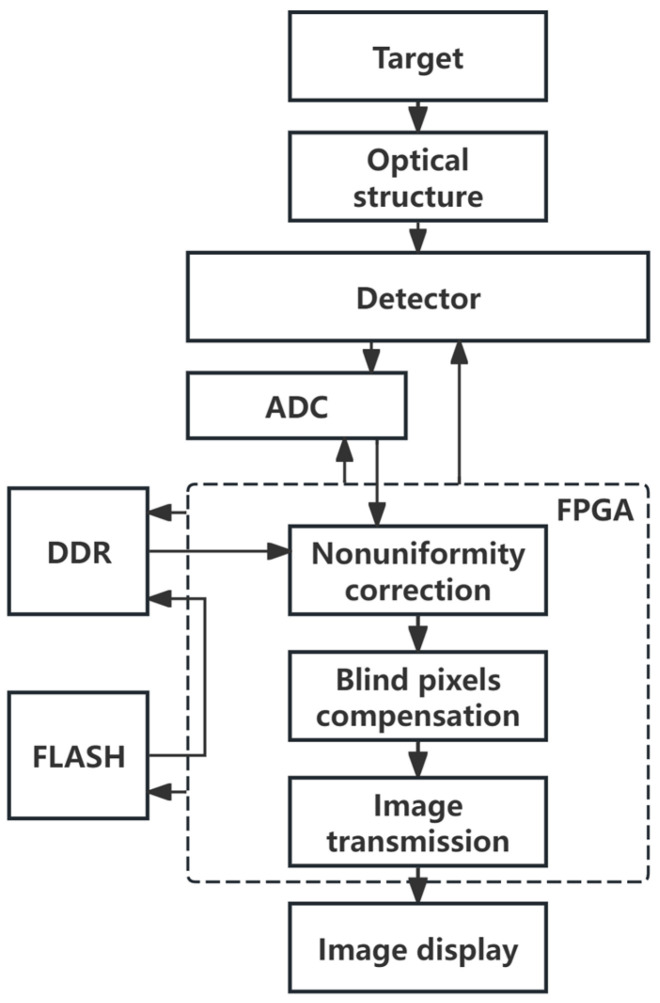
System implementation flowchart.

The overall implementation flowchart of the real-time non-uniformity correction system for high-frame-rate short-wave infrared images is shown in Figure 1.

The detector collects the target signal through the optical system. The FPGA controls the ADC to convert the analog signal output from the detector into digital DN values. Using the correction parameters read from DDR, the FPGA sequentially performs non-uniformity correction and blind pixel compensation on the DN value of each pixel and finally outputs the processed image.

### 3.1. Hardware System Design

**Figure 2:**
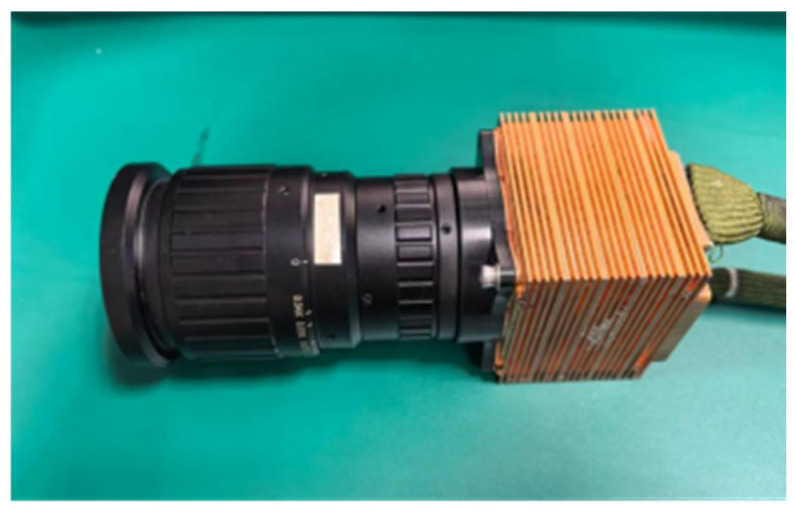
Physical pictures of the hardware of the experimental platform.

The hardware physical diagram of the experimental platform is shown in Figure 2.

The InGaAs short-wave infrared detector used in the system is developed by the Shanghai Institute of Technical Physics (SITP), Chinese Academy of Sciences, Shanghai, China. The detector has an area array format of 640 × 512, supports up to 8-channel parallel output, achieves a maximum frame rate of 1000 FPS, and supports thermoelectric cooler (TEC) cooling. In this experiment, the detector operates at a master clock frequency of 40 MHz, with data output via 8 channels, enabling a maximum frame rate of 800 FPS.

The analog-to-digital converter (ADC) selected is one that supports 8 channels, with a maximum sampling rate of 65 MSPS, 14-bit sampling precision, and serial Low-Voltage Differential Signaling (LVDS) output per channel, from Analog Devices, Wilmington, MA, USA. This provides excellent noise suppression and high-speed transmission performance.

The FPGA used is the XC7A100T-2FGG484I from Xilinx, San Jose, CA, USA, which handles image data acquisition, processing, and transmission, as well as driving and controlling peripheral hardware and communicating with the host computer.

For non-volatile storage, SPI NOR FLASH with a capacity of 256 Mb from Infineon (Neubiberg, Germany) is selected. Due to the limited on-chip RAM resources of the FPGA, FLASH is used to store the correction parameters, ensuring that the data is retained after power-off. The FLASH programming of correction parameters can be performed via the JTAG protocol or remotely updated through the UART protocol.

To meet the time requirements for reading correction parameters in a high-frame-rate system, DDR3 SDRAM memory from Micron (Boise, ID, USA) is also employed, with a total capacity of 2 Gb, a data bus width of 16 bits, and a maximum clock frequency of 933 MHz. In this experiment, the DDR operates at 400 MHz with a burst length set to 8.

The image transmission interface adopts Camera Link in Extended Full mode. This mode supports an 80-bit parallel data channel. At a transmission clock of 65 MHz, it provides a theoretical maximum throughput of 5.2 Gbps, which basically meets the real-time image transmission requirements of high-frame-rate imaging systems.

### 3.2. Design of the Real-Time Image Non-Uniformity Correction Software System

**Figure 3:**
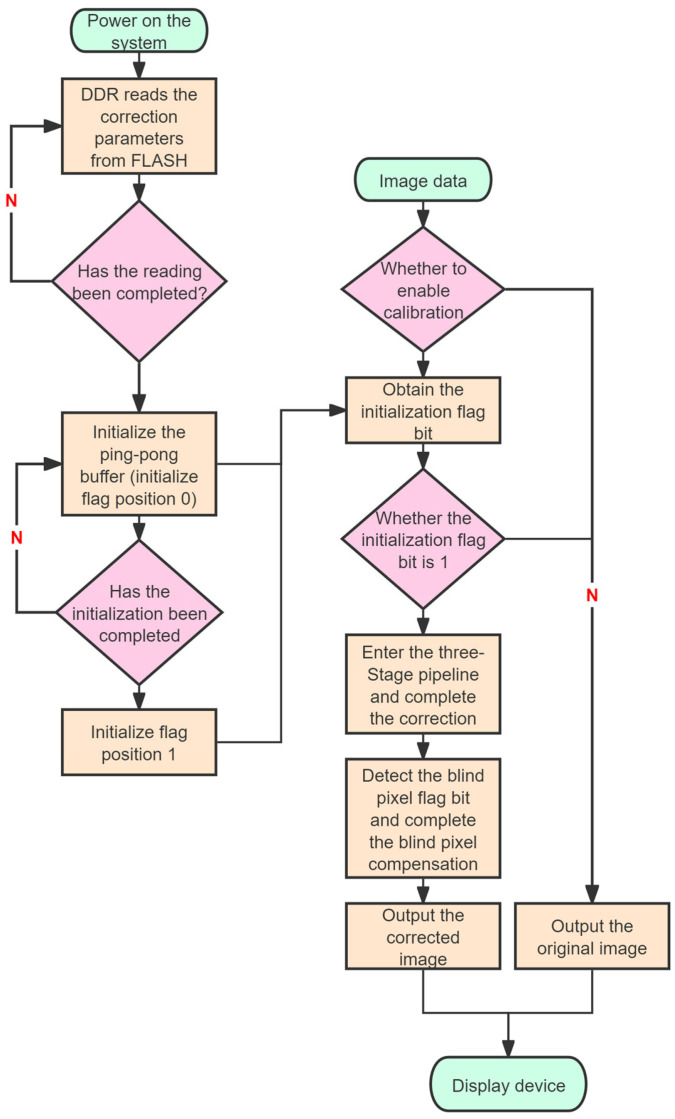
Real-time image correction software flowchart.

The software flowchart for real-time image correction is shown in Figure 3.

**Figure 4:**
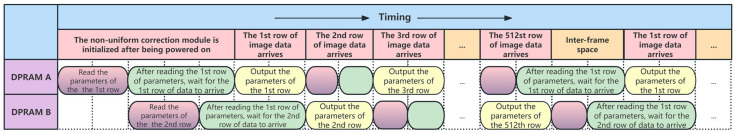
Ping-pong buffer reading DDR timing diagram.

Upon power-up of the system, the correction parameters stored in FLASH are automatically read into DDR. Once all correction parameters have been transferred to DDR, a ping-pong buffer is utilized to perform pre-read initialization for the correction parameters of the first two rows. This is necessary because DDR memory cells are capacitive, storing data as an electrical charge, and require frequent refresh and precharge operations to maintain data integrity. As a result, DDR read operations cannot be performed continuously in every clock cycle. If correction parameters were read directly from DDR and immediately used in the correction computation, it would be difficult to achieve precise alignment with the real-time image data stream, potentially causing misalignment between correction parameters and image data. To avoid this issue, a ping-pong buffer initialization mechanism is adopted. The initialization process and subsequent read operations are illustrated in Figure 4.

The ping-pong buffer consists of two alternately operating dual-port RAM (DPRAM): one DPRAM is used to read and store the correction parameters for the next row from DDR, while the other buffer provides the currently prepared correction parameters for use by the image data stream. This ping-pong buffer design ensures continuity and high efficiency in the parameter reading process. Moreover, the alternating operation of the DPRAMs effectively hides the access latency of DDR, significantly reducing overall waiting time.

As can also be seen from Figure 4, if the storage size of the correction parameters per row is too large, the time required to read one row becomes excessively long, which affects the subsequent parameter output. To continue satisfying the operational requirements of the ping-pong buffer, either the detector’s master clock frequency must be reduced to extend the arrival interval of image data which would lower the maximum output frame rate, or the DDR clock frequency must be increased to shorten the time needed to read one row of parameters. However, increasing the DDR clock frequency also increases system power consumption, raises requirements for hardware layout and routing, and may even necessitate the use of a more powerful FPGA to drive it. Therefore, the choice of 32-bit width for each correction parameter represents the optimal trade-off under the combined influence of multiple factors, including the FPGA model, correction performance, detector array size, detector master clock frequency, DDR data bus width, and effective DDR data rate.

Once the ping-pong buffer initialization is complete, the initialization flag bit is set to 1. When the host computer sends the command to enable correction and the initialization flag is detected as 0 after imaging starts, the system can only output raw image data. If the initialization flag is set to 1, real-time image processing operations are performed upon the arrival of image data. The real-time image data and the correction parameters read from the ping-pong buffer are fed into the pipeline module to perform the quadratic polynomial computation. The pipeline module adopts a three-stage pipeline design. Each pipeline multiplication operation utilizes four digital signal processing slices (DSP) resources. Since both the detector and the ADC output 8 channels, a total of 8 pipeline modules are instantiated, consuming 32 DSP resources in total. The addition operations are implemented using lookup tables (LUTs) and logic resources.

**Figure 5:**
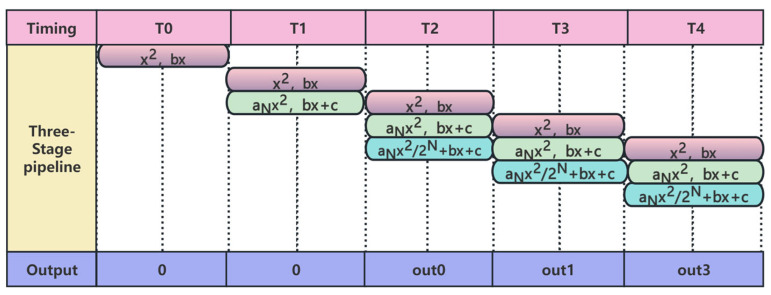
Three-stage pipeline timing diagram.

The timing diagram of the three-stage pipeline is shown in Figure 5. As can be observed from Figure 5, except for the first two clock cycles which have no valid output, every subsequent cycle produces one corrected output value. This demonstrates that the use of a pipeline significantly improves the overall system throughput. The adoption of pipelining not only fully utilizes the internal computational resources provided by the FPGA but also enables better timing convergence for the FPGA design.

After the pipeline computation is completed, the blind pixel flag bit in the correction parameters is checked. If the flag bit is 0, the pixel value remains unchanged. If the flag bit is 1, the 3 × 3 neighborhood mean compensation method is applied, in which surrounding normal pixels are used to replace the blind pixel for compensation. For boundary pixels (such as those in the first row, last row, first column, and last column) where the conventional 3 × 3 neighborhood cannot be applied, the FPGA employs dedicated judgment logic to specially handle these edge pixels by selecting only the valid neighboring pixels within the image to participate in the mean calculation. For clustered blind pixels, neighborhood mean compensation is generally inapplicable; therefore, the FPGA actively embeds manual compensation code. Finally, the image data processed through the image processing module is output to external display devices via the image transmission module. The FPGA resource utilization statistics for the correction processing module are shown in Table 2. As indicated in Table 2, after implementing the correction image processing module, the FPGA still has ample resource margin, which provides sufficient support for further iterative upgrades of system functions and the addition of other functional modules.

## 4. Experiments and Discussion

The evaluation method for the correction effect of infrared images can be performed using quantitative metrics such as Non-Uniformity (NU), by comparing the results before and after correction. The equation for calculating NU is as follows:

(9) $$\mathrm{NU}=\frac{1}{V_{avg}}\sqrt{\frac{1}{RC-d}\sum_{i=1}^{R}\sum_{j=1}^{C}{\left(V_{i,j}-V_{avg}\right)}^{2}}$$

where V_avg_ is the average response value of the effective pixels after excluding blind pixels, R is the number of rows in the image, C is the number of columns in the image, and d is the number of blind pixels.

The roughness index is a metric used to evaluate the smoothness of an image. A lower value indicates a smoother image and better noise correction performance. Its mathematical expression is:

(10) $$\rho =\frac{\left|\right|h*f|{|}_{1}+||h^{T}*f|{|}_{1}}{||f|{|}_{1}}$$

where f denotes the DN value of the image, the matrix h is the template [1, −1], h^T^ is the transpose of h, “*” represents the convolution operation, and ||⋅||_1_ denotes the L1 norm.

This experiment was conducted at an ambient temperature of 20 °C. The detector operating temperature was precisely stabilized at 20 °C using the TEC cooling system. To ensure uniform irradiance incidence, both the 15 sets of calibration data and the 8 sets of validation data were acquired through an integrating sphere. The obtained calibration and validation data basically cover the output dynamic range of the detector. During image acquisition, the detector integration time was set to 1 ms, and the required data were obtained by adjusting the lens aperture (f-number) and the irradiance conditions of the integrating sphere.

### 4.1. Performance Verification of the Improved Quadratic Polynomial Correction

**Figure 6:**
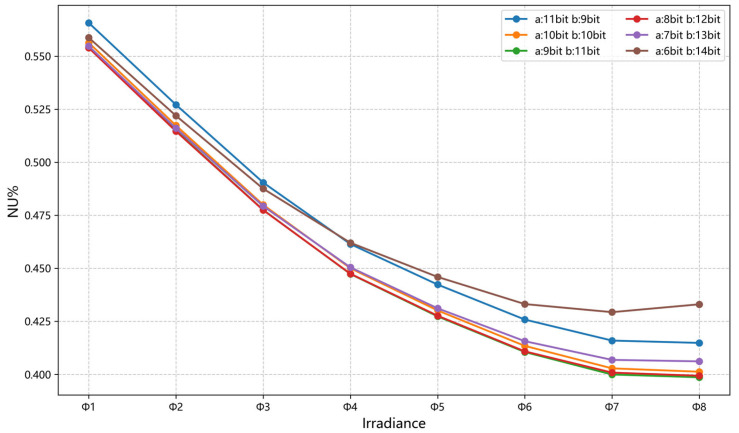
Correction performance of parameters with different bit widths on the validation set.

By performing least squares fitting on the calibration data collected under multiple groups of different irradiance levels, 640 × 512 sets of correction parameters are obtained, with a total storage size of 1280 KB. After the improvement, the storage size of the correction parameters is comparable to that of conventional two-point correction parameters. Based on the maximum and minimum values of the c parameter, a bit-width of 11 bits is allocated to c. The remaining 20 bits are dynamically allocated to the a_N_ and b parameters according to the correction performance. The correction effects under different bit-width allocations are shown in Figure 6.

**Figure 7:**
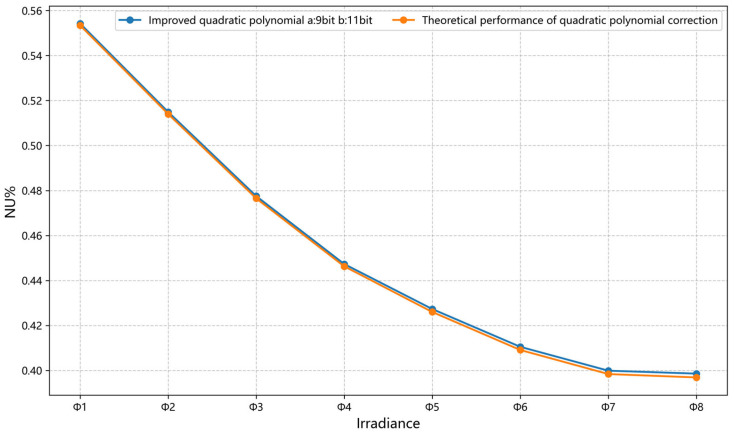
Comparison of the improved quadratic polynomial correction with theoretical performance.

The average NU of the validation data under different irradiance levels before correction was 2.8945%. As shown in Figure 6, when the bit-width of the a_N_ parameter is 9 bits and the bit-width of the b parameter is 11 bits, the overall correction performance is optimal, achieving an average NU of 0.4818 ± 0.0020% on the validation dataset after correction. As can be seen from Figure 7, compared with the theoretical performance curve of quadratic polynomial correction, the improved quadratic polynomial correction achieves performance on the validation data that is very close to the theoretical performance of quadratic polynomial correction. Theoretically, the average NU after quadratic polynomial correction is 0.4728 ± 0.0017%. Although the average NU of the improved method is 0.0090% higher, the paired t-test shows that the difference is statistically significant (*p* = 0.0144). Both methods exhibit extremely small standard deviations, indicating high stability and repeatability of the correction results. These results verify that the dynamic bit-width allocation and quantization strategy effectively balance hardware constraints and correction accuracy, achieving robust nonlinear compensation.

**Figure 8:**
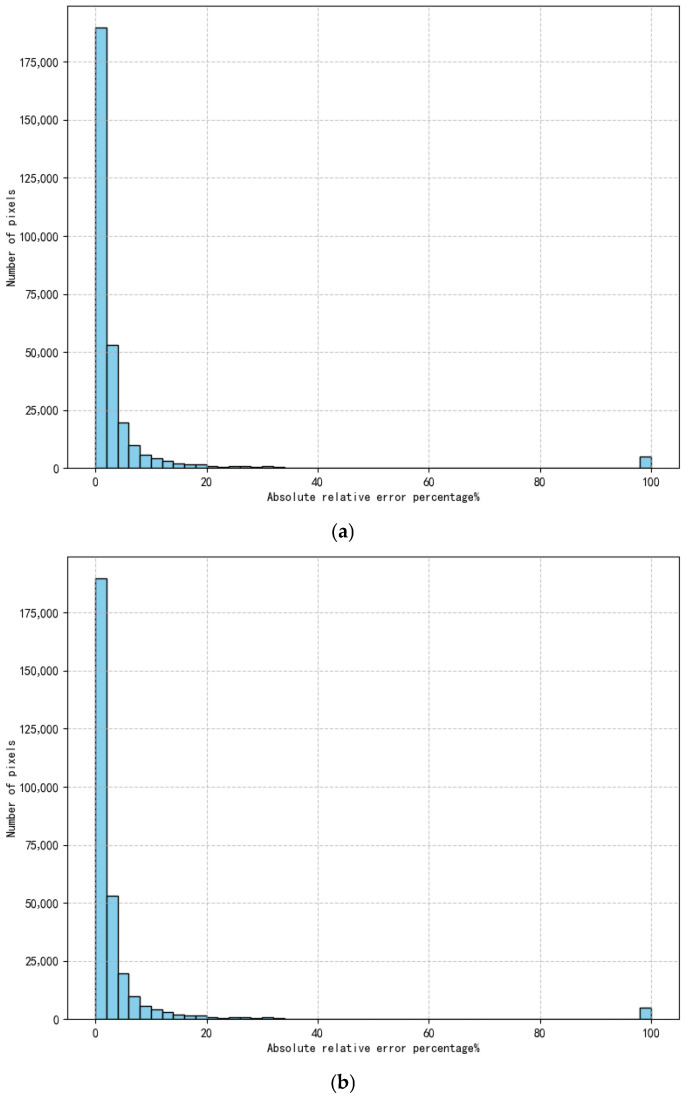

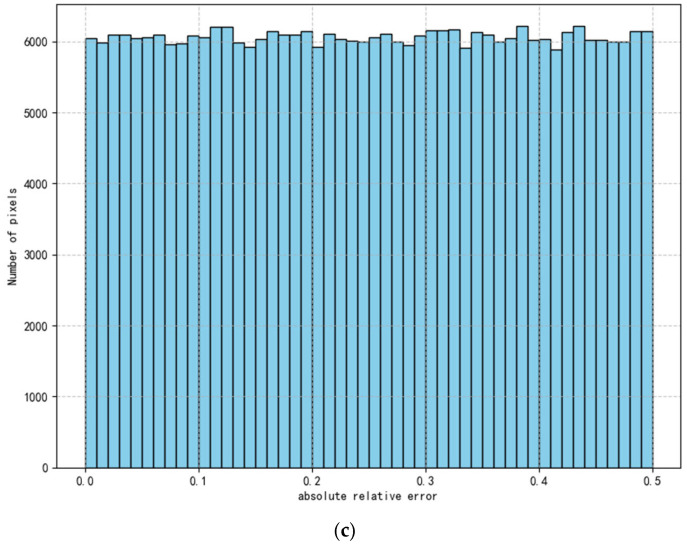
Histogram of parameter quantization error distribution: (**a**) histogram of absolute relative error percentage distribution for a_N_ parameter; (**b**) histogram of absolute relative error percentage distribution for b parameter; (**c**) histogram of absolute relative error distribution for c parameter.

Error analysis is performed on the quantized a_N_, b, and c parameters. The errors in a_N_ and b parameters mainly arise from the quantization error introduced during the conversion from floating-point to fixed-point representation [23]; the error in the c parameter primarily results from discarding the fractional part after rounding. The histograms of the errors for a_N_, b, and c parameters are shown in Figure 8.

As shown in Figure 8a, the absolute relative errors of the quantized a_N_ parameters for the vast majority of pixels are less than 6%. A small number of pixels exhibit absolute relative errors reaching 100%, which occurs because the a parameters of these pixels are very close to 0, making it impossible to represent a_N_ using fixed-point notation with the limited bit-width. In this experiment, the bit-width for quantizing a_N_ is 9 bits and N is 16, so the minimum representable resolution is 2^−24^. The effective bit-width for the pixel is 14 bits with the quantized DN value ranging from 0 to 16,383. Under different irradiance levels, when a is 2^−24^, the contribution of ax^2^ to the pixel DN value is approximately in the range [0, 0.0976%]. For the pixels in this experiment where the absolute relative error of a_N_ reaches 100%, their a parameters are smaller than 2^−24^, resulting in the contribution of the ax^2^ term being less than [0, 0.0976%], which has only a limited impact on the overall correction accuracy. As can be seen from Table 3, when the quantization bit-widths of the b and c parameters remain unchanged, the absolute relative error introduced by quantization gradually increases as the bit-width of a_N_ decreases, resulting in a continuous decline in correction performance. Although the quadratic term ax^2^ accounts for only a small proportion in the pixel DN value, excessive loss of quantization precision still exerts a significant impact on the overall correction performance of the algorithm.

As can be seen from Table 4, when the quantization bit-widths of the a_N_, b, and c parameters remain unchanged, the absolute relative error introduced by quantization gradually increases as the magnification factor N decreases, resulting in a continuous decline in correction performance. When the magnification factor is reduced to 8, the quantization error reaches as high as 99.99%, at which point parameter a becomes almost ineffective. Combined with the results in Table 3, it can be seen that introducing the magnification factor effectively reduces the bit-width requirement for parameter a and significantly improves bit-width utilization under limited bit-width conditions.

From Figure 8b, it can be observed that the absolute relative errors of the quantized b parameters for most pixels are less than 0.06%, indicating high quantization precision. During the correction process, since the b parameter values are close to 1, the term bx occupies a very large proportion of the corrected pixel DN value. Combined with Figure 6, it is evident that, within a certain range, increasing the quantization bit-width of the b parameter to improve quantization precision can significantly enhance the correction performance. Therefore, the quantization precision of the b parameter has a substantial influence on the overall correction performance. In practical applications, the bit-width allocation priority for the b parameter should be higher than that for a_N_ and c parameters. As can be seen from Table 5, when the quantization bit-widths of the a_N_ and c parameters remain unchanged, the absolute relative error introduced by quantization gradually increases as the bit-width of the b parameter decreases, resulting in a continuous decline in correction performance. Since the linear term bx occupies a large proportion in the pixel DN value, when the quantization bit-width of b is reduced to 5 bits, the absolute relative error is only 2.44%, yet the corrected non-uniformity results show that the b parameter becomes almost ineffective at this point. Therefore, the quantization precision of the b parameter has a significant influence on the overall correction performance.

Since the c parameter is quantized using a signed integer representation with the fractional part rounded to the nearest integer, the resulting error is very small. As shown in Figure 8c, all quantization errors for the c parameter are less than or equal to 0.5, and this level of error has a negligible impact on correction accuracy.

Although the limitation of parameter quantization bit-width increases the quantization error of the parameters, the correction performance of the proposed method is still superior to that of two-point correction and multi-point correction. As shown in Table 6, a quantitative comparison is conducted among the raw image (Raw) and six different non-uniformity correction methods, including conventional two-point correction, two-point two-segment correction, two-point four-segment correction, two-point six-segment correction, two-point eight-segment correction, and the improved quadratic polynomial correction method proposed in this paper (Ours). The results show that as the number of segments increases, both the NU and ρ of the two-point multi-segment correction methods improve gradually. Among them, the two-point eight-segment correction achieves relatively good performance, with an NU of 0.4891% and a ρ of 1.05%. However, the improved quadratic polynomial correction method proposed in this paper further improves both NU and ρ, reducing them to 0.4818% and 0.98%, respectively, outperforming the two-point eight-segment correction.

Compared with conventional two-point and multi-segment correction methods, the main advantages of the improved quadratic polynomial correction lie in the following aspects: the traditional two-point multi-segment methods are essentially based on piecewise linear correction and approximate the real response curve by increasing the number of segments. Although they can effectively reduce non-uniformity, they tend to produce step errors at segment boundaries, and a larger number of parameters increases hardware resource consumption and parameter readout time. In contrast, the improved quadratic polynomial correction proposed in this paper introduces a quadratic polynomial model and applies targeted optimization to the model parameters, enabling a smoother and more accurate fitting of the detector’s nonlinear response curve. This not only effectively suppresses fixed pattern noise but also avoids the step errors commonly seen in piecewise linear methods, thereby improving the overall smoothness and visual quality of the corrected image. The experimental results fully verify the superiority of the proposed method over conventional two-point and multi-point correction approaches.

**Figure 9:**
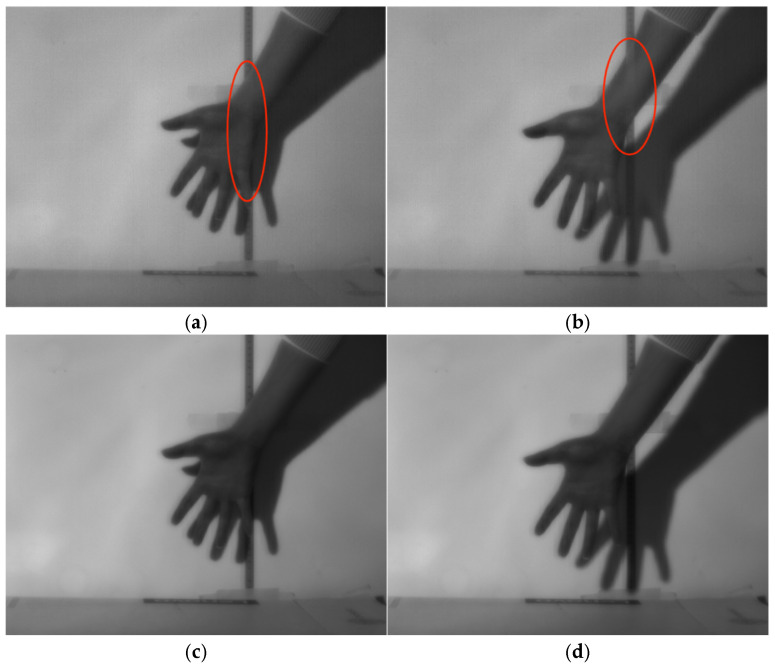
Comparison of the THPF algorithm with improved quadratic polynomial correction: (**a**) The 10th frame image processed by the THPF algorithm; (**b**) The 50th frame image processed by the THPF algorithm; (**c**) The 10th frame image processed by the proposed method; (**d**) The 50th frame image processed by the proposed method.

The THPF algorithm is one of the widely used scene-based non-uniformity correction methods. Figure 9a,b show the image results after THPF processing. The images contain a moving hand and a ruler fixed on the wall. The red-marked region exhibits the typical “ghosting artifact” phenomenon commonly seen in scene-based non-uniformity correction methods. This artifact mainly arises from the lag in algorithm convergence, causing the texture information of the stationary ruler in the background to be erroneously superimposed onto the moving hand. This phenomenon is particularly evident in scenes where the target is moving while the background remains relatively static. Figure 9c,d present the image results after applying the improved quadratic polynomial correction method. It can be observed that the processed images are significantly smoother overall, with no obvious grid-like patterns.

As shown in Table 7, although the NU and ρ of the image are improved to some extent after THPF processing, the presence of ghosting artifacts leads to a noticeable subjective degradation in overall image quality. Although the ghosting phenomenon has been effectively suppressed to a certain degree with the continuous development and iteration of related algorithms [24], the increased algorithm complexity also significantly raises the demand for computational resources, thereby imposing higher requirements on the hardware platform. Some methods even rely on high-performance computing devices such as Graphics Processing Unit (GPU) to achieve real-time processing [25].

### 4.2. Implementation of High-Frame-Rate Real-Time Non-Uniformity Correction Based on an FPGA

Test results show that the processing time of the FPGA for one frame of a 640 × 512 image is approximately 1.24 ms, and the measured maximum output frame rate reaches 800 FPS.

**Figure 10:**
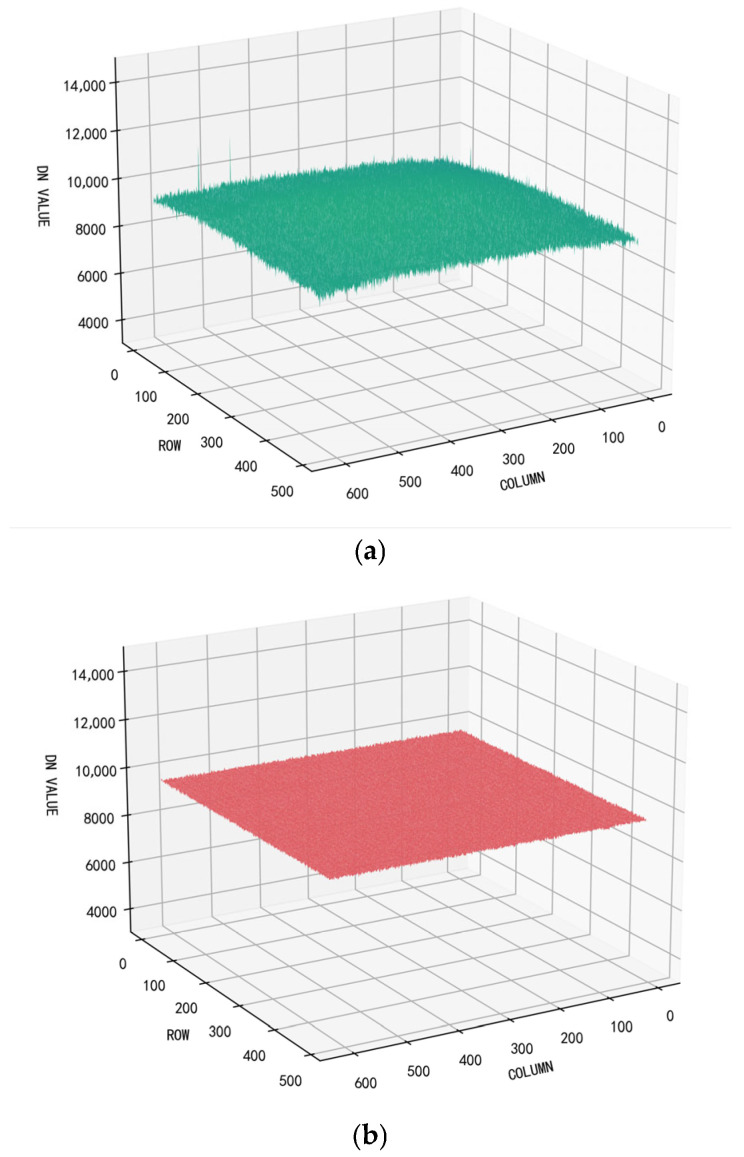
Improved quadratic polynomial correction 3D views of short-wave infrared images before and after correction: (**a**) image before correction; (**b**) improved quadratic polynomial correction (FPGA implementation).

From Figure 10a, it can be observed that the surface of the 3D image is extremely rough, which is a manifestation of non-uniformity; the prominent “spikes” correspond to blind pixels in the image.

Figure 10b shows the result after applying the improved quadratic polynomial correction implemented on the FPGA. It is clearly evident that the DN values of each pixel become nearly identical after correction, the image surface becomes smooth, blind pixels are effectively compensated, and the protruding “spikes” are eliminated. As can be seen from Table 8, after enabling correction, both the non-uniformity and roughness of the image are significantly reduced.

**Figure 11:**
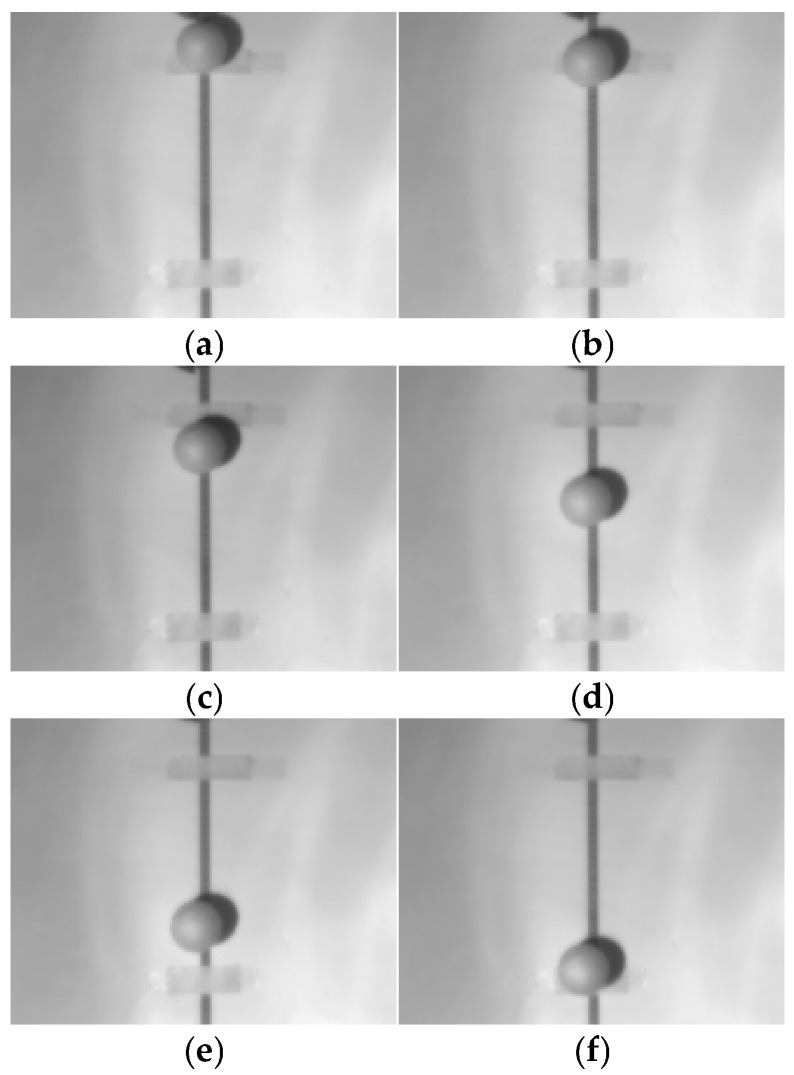
Verification of high-frame-rate image processing capability: (**a**) Frame 1 (T0); (**b**) Frame 59 (T0 + 72.5 ms); (**c**) Frame 99 (T0 + 122.5 ms); (**d**) Frame 139 (T0 + 172.5 ms); (**e**) Frame 179 (T0 + 222.5 ms); (**f**) Frame 192 (T0 + 238.75 ms).

Verification of the high-frame-rate image processing capability was conducted. A freely falling small ball was used as the target, and the falling process was captured. The imaging effect after image processing is shown in Figure 11. The figure clearly illustrates the process of the small ball in free fall. According to the free-fall distance calculation equation:

(11) $$S=\frac{1}{2}gt^{2},$$

The capture frame rate is 800 FPS. Based on the frame interval, the time is determined to be 0.23875 s, with a falling distance of 0.28 m. Using these values, the gravitational acceleration g is calculated to be approximately 9.82 m/s^2^. After correction, the image shows no noticeable grid-like patterns, appears smooth overall, and blind pixels have been effectively compensated. This fully demonstrates the system’s capability for real-time non-uniformity correction at high frame rates.

## 5. Conclusions

The real-time non-uniformity correction method for high-frame-rate short-wave infrared images proposed in this paper improves the quadratic polynomial correction based on FPGA implementation. The storage size of the correction parameters is comparable to that of conventional two-point correction parameters. While performing non-uniformity correction, it simultaneously achieves blind pixel compensation. Without requiring additional hardware upgrades, the correction performance surpasses that of two-point correction and approaches or even exceeds that of two-point eight-segment correction. The real-time non-uniformity correction processing achieves a maximum frame rate of 800 FPS.

Although the proposed method offers certain advantages, it still has some limitations. First, the method relies on a calibration process and cannot completely eliminate long-term non-uniformity drift caused by temperature changes or device aging. Second, the calibration process requires a large amount of data, and the quantization of correction parameters along with the dynamic bit-width allocation process is relatively complex, which is unfavorable for subsequent parameter updating and maintenance. Furthermore, limited by the expressive capability of the quadratic model itself, the quadratic polynomial may not accurately fit the response characteristics of detectors with poor linearity.

Future work may consider combining the proposed calibration-based method with scene-based adaptive algorithms to construct a hybrid correction framework, thereby improving the long-term stability of the system. In addition, the current model can be extended to higher-order forms to more accurately fit the detector’s response curve. Further extending the method to larger-scale detector arrays or applying it to higher-frame-rate imaging systems also represents a valuable research direction.

## Figures and Tables

**Table 1:** Arrangement of correction parameters for each pixel.

	Blind Pixel Flag	a_N_	b	c
Bit-Width Occupation	1	a_bit	b_bit	c_bit
Bit Position	The 31st bit	The 30th to (31-a_bit)th bit	The (30-a_bit)th to (31-a_bit-b_bit)th bit	The (c_bit-1)th to 0th bit

**Table 2:** FPGA resource usage statistics.

Resource Type	Used	Available	Utilization
Registers	6384	126,800	5.03%
LUTs	8780	63,400	13.85%
RAM	28	135	20.74%
DSPs	32	240	13.33%

**Table 3:** The influence of the quantization bit-width of a_N_ on correction performance.

The Quantization Bit-Width of a_N_	NU	Absolute Relative Error Percentage
9	0.4818%	4.88%
8	0.4909%	14.97%
7	0.5331%	42.00%
6	0.9890%	90.68%

**Table 4:** The influence of the magnification factor N on correction performance.

The Magnification Factor N	NU	Absolute Relative Error Percentage
16	0.4818%	4.88%
14	0.4909%	14.97%
12	0.5331%	42.00%
10	0.9890%	90.68%
8	1.5229%	99.99%
6	1.5229%	99.99%

**Table 5:** The influence of the quantization bit-width of b on correction performance.

The Quantization Bit-Width of b	NU	Absolute Relative Error Percentage
11	0.4818%	0.02%
10	0.4855%	0.05%
9	0.4911%	0.10%
8	0.5364%	0.21%
7	0.7071%	0.44%
6	1.2324%	0.99%
5	2.4831%	2.44%

**Table 6:** Comparison of the improved quadratic polynomial correction with two-point correction and multi-point correction.

Method	NU	ρ
Raw	2.8945%	3.17%
Two-point	0.5930%	1.14%
Two-point two-segment	0.5185%	1.10%
Two-point four-segment	0.5096%	1.08%
Two-point six-segment	0.4987%	1.07%
Two-point eight-segment	0.4891%	1.05%
Ours	0.4818%	0.98%

**Table 7:** Comparison of evaluation metrics before and after THPF algorithm and improved quadratic polynomial correction.

Method	NU	ρ
Raw	23.88%	3.19%
THPF	23.03%	2.66%
Ours	20.98%	1.37%

**Table 8:** Comparison of evaluation metrics before and after the improved quadratic polynomial correction implemented on an FPGA.

Type	NU	ρ
Raw	2.8651%	3.29%
After correction	0.4867%	0.95%

## Data Availability

The data presented in this study are available upon request from the corresponding author.

## References

[1] Zhang J., Zhong S., Li Z., Mao W., Chen N., Bai P., Han Q., Yao L. (2021). High Frame Rate 384 × 288 Long-Wave Digital Infrared Focal Plane Array Detector. Acta Opt. Sin..

[2] Fu P., He D., Liu J., Wang Y. (2025). High-precision spot centroid positioning of high-frame-rate short-wave infrared images for satellite laser communication. Infrared Millim. Waves.

[3] Schulz M., Caldwell L. (1995). Nonuniformity correction and correctability of infrared focal plane arrays. Infrared Phys. Technol..

[4] Milton A., Barone F., Kruer M. (1985). Influence of Nonuniformity on Infrared Focal Plane Array Performance. Opt. Eng..

[5] Feng L., Liu S., Zhao K., Guan A. (2006). Method of Nonuniformity Correction for Irfpa with Nonlinear Response. Infrared Millim. Waves.

[6] Cheng G., Chen X., Wang X., Wang J. (2013). Information Acquisition System for Thermal Infrared Focal Plane Array with High Frame-rate and Low-noise. Infrared Technol..

[7] Wang C., Wang C., Gu J., Zhao X. (2022). An improved non-uniformity correction algorithm based on calibration. Chin. Opt..

[8] Han L., Li G. (2023). An improved non-uniformity correction algorithm based on calibration. Ship Electron. Eng..

[9] Wang H. (2008). Fast Nonuniformity Correction Algorithm Based on Cubic Polynomial Fitting. Aero Weapon..

[10] Wang Y., Chen J., Liu Y., Xue Y. (2003). Study on Two-Point Multi-Section Irfpa Nonuniformity Correction Algorithm. Infrared Millim. Waves.

[11] Yao Q., Gu G. (2012). Study of nonuniformity correction precision of IRFPA. Infrared.

[12] Dai S., Zhang T. (2007). Improvement on Multi-point Correction Method of IRFPA. Infrared Technol..

[13] Su Y., Su J., Liu C., Su L., Hu Z., Yang Z. (2014). Scene-based NUC Algorithms for Domestic-made IR Detector. Infrared Technol..

[14] Zhang T., Shi C., Li J., Liu H., Yuan Y., Zhou Y. (2007). Overview of Research on the Adaptive Algorithms for Nonuniformity Correction of Infrared Focal Plane Array. Infrared Millim. Waves.

[15] Harris J., Chiang Y. (1999). Nonuniformity Correction of Infrared Image Sequences Using the Constant-Statistics Constraint. IEEE Trans. Image Process..

[16] Wang D., Ren Z., Liu S., Zhao Y., Fang H., Zhang L. (2021). Polarization redundancy estimation scene-based non-uniformity correction algorithm. Infrared Millim. Waves.

[17] Sheng Y., Dun X., Qiu S., Li L., Jin W., Wang X. (2021). On-orbit non-uniformity correction method for infrared remote sensing systems using controllable internal calibration sources. Infrared Millim. Waves.

[18] Hardie R., Baxley F., Brys B., Hytla P. (2009). Scene-Based Nonuniformity Correction with Reduced Ghosting Using a Gated LMS Algorithm. Opt. Express.

[19] Hardie R., Hayat M., Armstrong E., Yasuda B. (2000). Scene-Based Nonuniformity Correction with Video Sequences and Registration. Appl. Opt..

[20] Huang Y., Zhang B., Wu J., Chen Y., Ji L., Wu X., Yu S. (2020). Adaptive multipoint calibration non-uniformity correction algorithm. Infrared Technol..

[21] Huang Y., Huang H. (2024). Shutter-less Non-uniformity Correction Methods in Uncooled Infrared Imagery. J. Electron. Inf. Technol..

[22] Zheng H., Qiu X., Zhang H., Huang M. (2023). Design of FPGA-Based Non-Uniformity Correction System for Infrared Images. Infrared.

[23] Shi Y., Zhang T., Wang Y., Jiang H. (2005). Study on Two-points Nonuniformity Correction Algorithm for IRFPA and Implementation by Using FPGA. Laser Infrared.

[24] Li M., Wang Y., Sun H. (2023). Single-Frame Infrared Image Non-Uniformity Correction Based on Wavelet Domain Noise Separation. Sensors.

[25] Li T., Zhao Y., Zhou G. (2021). Non-Uniformity Correction of Infrared Images Based on Improved CNN with Long-Short Connections. IEEE Photonics J..

